# Tubulointerstitial nephritis complicating IVIG therapy for X-linked agammaglobulinemia

**DOI:** 10.1186/1471-2369-15-109

**Published:** 2014-07-08

**Authors:** Keisuke Sugimoto, Hitomi Nishi, Tomoki Miyazawa, Norihisa Wada, Akane Izu, Takuji Enya, Mitsuru Okada, Tsukasa Takemura

**Affiliations:** 1 Department of Pediatrics, Kinki University Faculty of Medicine, 377-2 Ohno-higashi, 589-8511 Osaka-Sayama, Japan

**Keywords:** Bruton agammaglobulin tyrosine kinase (BKT) gene, Immune complexes, Steroid therapy, Tubular deposition, Tubular atrophy

## Abstract

**Background:**

Patients with X-linked agammaglobulinemia (XLA) develop immune-complex induced diseases such as nephropathy only rarely, presumably because their immunoglobulin (Ig) G concentration is low. We encountered a patient with XLA who developed tubulointerstitial nephritis during treatment with intravenous immunoglobulin (IVIG).

**Case presentation:**

A 20-year-old man was diagnosed with XLA 3 months after birth and subsequently received periodic γ-globulin replacement therapy. Renal dysfunction developed at 19 years of age in association with high urinary β2-microglobulin (MG) concentrations. A renal biopsy specimen showed dense CD3-positive lymphocytic infiltration in the tubulointerstitium and tubular atrophy, while no IgG4-bearing cell infiltration was found. Fibrosclerosis and crescent formation were evident in some glomeruli. Fluorescent antibody staining demonstrated deposition of IgG and complement component C3 in tubular basement membranes. After pulse steroid therapy was initiated, urinary β2-MG and serum creatinine concentrations improved.

**Conclusion:**

Neither drug reactions nor collagen disease were likely causes of tubular interstitial disorder in this patient. Although BK virus was ruled out, IgG in the γ-globulin preparation might have reacted with a pathogen present in the patient to form low-molecular-weight immune complexes that were deposited in the tubular basement membrane.

## Background

In X-linked agammaglobulinemia (XLA), mutation involving the Bruton agammaglobulin tyrosine kinase (BTK) gene induces a B-cell maturation disorder that causes congenital immunodeficiency in which repeated bacterial infections reflect antibody production failure [1,2]. Prognosis for survival is relatively favorable owing to immunoglobulin replacement therapy (intravenous immunoglobulin therapy, or IVIG) [3].

We encountered a patient with BTK gene mutation (p.Gln412X)-induced XLA who developed renal dysfunction associated with increased urinary β2-microglobulin during IVIG therapy. Renal histology indicated a tubular interstitial disorder. Glomerular immune complex deposition such as in membranous nephropathy [4] and membranoproliferative glomerulonephritis [5] occasionally has been reported in association with IVIG therapy for XLA. To our knowledge, however, tubulointerstitial nephritis (TIN) showing immune complex deposition in the tubular basement membrane has not previously been reported in XLA patients receiving IVIG therapy.

## Case presentation

A 20-year-old man who was diagnosed with XLA 3 months after birth was treated periodically with IVIG. Mild renal dysfunction developed at 19 years. Serum creatinine (Cr) and blood urea nitrogen (BUN) were 1.5 and 30 mg/dL respectively, and urinary β2-microglobulin was elevated. The patient was admitted to our department for further evaluation and treatment.

**Figure 1:**
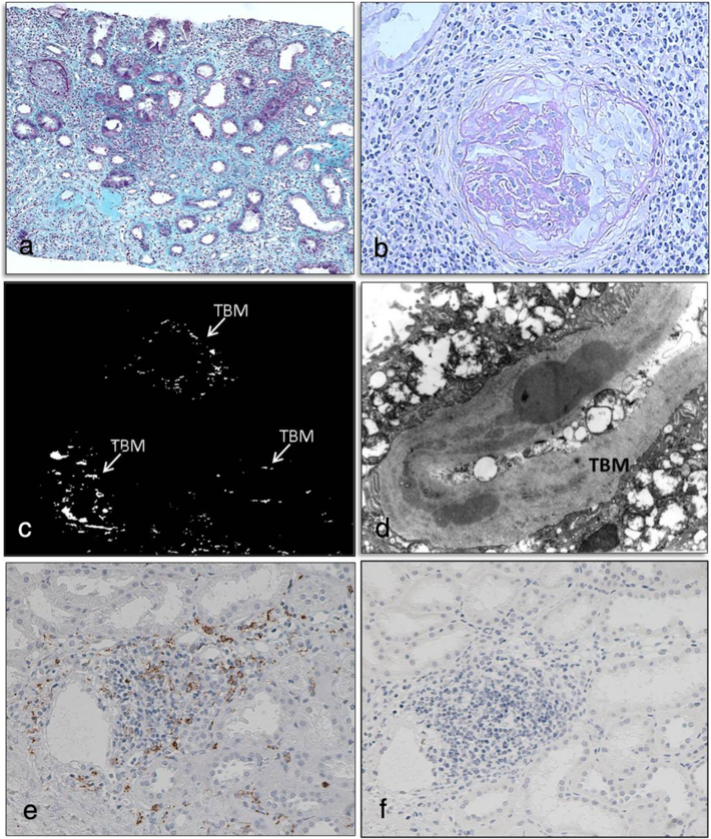
**Renal histologic findings in the patient.** Marked tubulointerstitial mononuclear cell infiltration was observed (**a**; Masson trichrome stain, ×100). Crescent formation was noted in some glomeruli (**b**; periodic acid-Schiff stain, ×200). Fluorescent antibody staining demonstrated granular deposition of IgG in the tubular basement membrane (**c**; ×200). Electron-dense deposits were present in the tubular basement membrane (**d**; original magnification, ×6000). Immune cells infiltrating in tubulointerstitial tissue were mainly CD3-positive T-cells (**e**), as opposed to IgG4-bearing cells (**f**; ×200).

Urinalysis on admission showed specific gravity of 1.017, 1+ qualitative protein, and 0.14 g/day quantitative protein. Microscopy showed 1 to 4 red blood cells per high-power field (HPF). White blood cells were 1 to 4 per HPF. Urine β2-microglobulin was 32550 μg/day (normal, below 250), and N-acetyl-β-D-glucosaminidase was 17.9 U/L (normal, 0.3 to 11.5). Creatinine clearance was 39.2 mL/min/1.73 m^2^ (normal, 65 to 142). The findings suggested tubular interstitial disorder causing renal dysfunction. No uveitis was detected. On hematologic examination, the red blood cell count was 548 × 10^4^ /μL; white blood cell count, 8400 /μL; platelet count, 15.5 × 10^4^/μL; and erythrocyte sedimentation rate, 4 mm/hour. In serum, Na was 142 mEq/L; K, 3.9 mEq/L; C-reactive protein, 2.8 mg/dL; BUN, 30 mg/dL; Cr, 1.29 mg/dL; and cystatin C, 1.96 mg/L (normal, 0.56 to 0.87). In sum, inflammatory markers were mildly elevated and moderate renal dysfunction was present. The IgG concentration was 685 mg/dL (normal, 870 to 1700 mg/dL); and IgM, below 20 mg/dL (normal, 33 to 190 mg/dL). Complement components were normal. Serum IgG4 concentration was below 1% of total serum IgG concentration. All autoantibodies were absent (antinuclear, anti-DNA, rheumatoid arthritis hemagglutination antibodies, anti-cyclic citrullinated peptide, anti-SS-A/Ro, anti-SS-B/La, and myeloperoxidase-ANCA). On investigation for pathogens, cytomegalovirus pp65 antigen, anti-VCA IgM antibody, and γ-interferon specific for tuberculosis were not detected. Adenovirus, herpes simplex virus, and BK virus were not detected by real-time polymerase chain reaction. Lymphocyte stimulation tests (DLST) with D-mannitol, glycine, and polyethylene glycol (all components of the patient’s γ-globulin preparation) were negative. No physical, laboratory, or radiologic findings suggesting Castleman disease or malignant lymphoma were demonstrated.Examination of renal biopsy specimen showed marked mononuclear cell infiltration in the interstitium (Figure 1a), and loss of tubular epithelial cells, cloudy degeneration, and irregularity of the basement membrane in some renal tubules. Some glomeruli showed cellular crescent formation (Figure 1b) and fibrosclerotic lesions. Fluorescent antibody staining detected granular depositions of IgG (Figure 1c) and complement component C3 in the tubular basement membrane. By electron microscopy (Figure 1d), electron-dense deposits were observed in the tubular basement membrane. Immune cells infiltrating in the tubulointerstitium were predominantly CD3 antigen-positive lymphocytes (T-cells; Figure 1e), but not IgG4-bearing cells (Figure 1f).

Steroid therapy was administered including methylprednisolone pulse therapy followed by prednisolone (PSL). Urinary findings and renal function slowly improved, and progress continued after gradual dose reduction. Although the patient still is receiving IVIG therapy, urinary abnormalities and renal function deterioration have not recurred.

## Discussion

Immune-comples induced nephritis and complement-associated renal impairment are infrequent in XLA patients, most likely because of their very low IgG concentrations [6]. We encountered a young man who developed tubulointerstitial nephritis (TIN) during IVIG for XLA. Glomerular immune complex deposition related to membranous nephropathy [4] and membranoproliferative glomerulonephritis [5] during the course of XLA have been reported occasionally. Nephrotic syndrome with TIN during IVIG therapy for secondary hypogammaglobulinemia during childhood has been described [7]. However, the reporting authors concluded that TIN might have been caused by a local allergic reaction, considering a predominance of eosinophils among cells infiltrating the tubulointerstitum. To our knowledge, however, TIN induced by immune complex deposition in the renal tubules, such as that occurring in our patient, has not been reported previously.

In XLA, viral infections follow a normal course except for enterovirus infection, in which effective host defense requires antibodies. XLA patients are vulnerable to this infection, which may become persistent and progressive [8]. In our patient, IgG and complement deposits were demonstrated in the tubular basement membrane by fluorescent antibody staining and electron microscopy, suggesting several possible pathogenetic mechanisms. Complement may have been activated locally after immune complex formation by interactions between an antigen present in the patient and the IgG contained in the γ-globulin preparation, inducing a tubular interstitial disorder. However, no specific infection could be identified in our patient. Although tubulointerstitial immune-complex nephritis is a very rare form of TIN, such nephritis can be caused by treatments such as nonsteroidal anti-inflammatory drugs [9], by autoimmune diseases such as lupus erythematosus [10], or IgG4-related diseases [11]. However, our patient received no long-term drug treatment apart from IVIG, and had no collagen disease or IgG4-related disease according to repeated laboratory examinations.

Wegmuller et al. [12] reported that complement activation may occur in both healthy individuals and patients with congenital humoral immunodeficiency. A few B cells persist in XLA patients, producing a small amount of IgG in some patients [13]. The exogenous, non-self IgG infused in IVIG might have induced autoantibody production against exogenous IgG with consequent nephropathy, but this mechanism cannot be proven in our patient; because to prevent fatal infection, he received periodic doses of IVIG, rendering measurement of self-produced IgG impossible. When type III allergy is involved in disease development and the antigen concentration exceeds the antibody concentration, immune complexes will have low molecular weight and may well be deposited outside the tubular basement membrane [14]. Alternatively, antigen concentrations may be excessive in XLA patients, with low-molecular-weight immune complexes formed in the circulation possibly passing through the glomeruli to enter the urinary space and then deposited in the tubular basement membrane.

## Conclusions

In patients with congenital humoral immunodeficiency patients, vigilance is needed to detect development of renal dysfunction during IVIG therapy. Not only glomerular disease but also TIN may occur, as occurred in our patient.

## Consent

Written informed consent was obtained from the patient for publication of this case report and any accompanying images.

## Abbreviations

XLA: X-linked agammaglobulinemia; IVIG: Intravenous immunoglobulin therapy; Ig: Immunoglobulin; BKT: Burton agammaglobulin tyrosine kinase; DLST: Lymphocyte stimulation tests; TIN: Tubulointerstitial nephritis.

## Competing interests

The authors declare that they have no competing interests.

## Authors’ contributions

KS, HN, TM, AI, NW, ET, and MO were the attending physicians of this patient. TT was responsible for the design of this case report, and manuscript write-up. There were no “ghost writers”. All authors read and approved the final manuscript.

## Pre-publication history

The pre-publication history for this paper can be accessed here:

http://www.biomedcentral.com/1471-2369/15/109/prepub
